# Effect of greenness on asthma in children: A systematic review

**DOI:** 10.1111/phn.12701

**Published:** 2020-01-03

**Authors:** Kim Hartley, Patrick Ryan, Cole Brokamp, Gordon L. Gillespie

**Affiliations:** ^1^ University of Cincinnati College of Nursing Cincinnati OH USA; ^2^ Division of Biostatistics and Epidemiology Cincinnati Children's Hospital Medical Center Cincinnati OH USA; ^3^ Department of Pediatrics University of Cincinnati College of Medicine Cincinnati OH USA

**Keywords:** child, environment, green space, greenspace, nature, pediatric, respiratory, urban health

## Abstract

Greenness such as trees, plants, and shrubs may positively influence mental and physical health, but the relationship between greenness and asthma is poorly understood. Because asthma is the most prevalent child respiratory disease internationally, elucidating the role of greenness may substantially benefit public health. The purpose of this systematic review was to synthesize findings related to effects of greenness on asthma in children. Following PRISMA guidelines, six databases were searched for international publication of primary research results relevant to the relationship between greenness and child asthma. Of 82 initial results, seven articles remained after removal of duplicates and applying exclusion criteria. Six reported no direct association between greenness and child asthma, while one found increased greenness protective for asthma. None found a negative direct association between greenness and child asthma. Evidence supported benefits of greenness on child asthma through mediation of factors such as exposure to tobacco smoke, high traffic volume, and difficult family relationships. Even without a direct association, greenness can be considered a public health asset as it may mediate other factors contributing to asthma in children. Public health nurses can use these findings to educate clients and partners while advocating for policies to protect greenness.

## 1 BACKGROUND

Public health nurses are positioned to evaluate environments in which people live, learn, work, and play (American Public Health Association [APHA], 2013), understanding that health is affected by physical features and exposures within the environment (Centers for Disease Control & Prevention [CDC], 2014). One feature influencing health directly or through mediation of other exposures is environmental greenness.

Greenness is vegetation such as trees, grasses, and plants that may be planned (e.g., parks, gardens, street trees) or unplanned (e.g., existing forested areas). The relationship between greenness and health is an emerging construct in environmental and public health. Findings have shown promise for benefits of greenness on multiple physical and mental health outcomes, including stress and headache reduction, improvement in cardiovascular and respiratory health, longevity among the elderly, cognitive restoration, increased self‐discipline, reduced aggression, improved birth outcomes, reduced crime and interpersonal violence, and increased social ties and quality of life (Hartig, Mitchell, de Vries, & Frumkin, 2014; Nieuwenhuijsen et al., 2014; Villeneuve et al., 2012). Some potential pathways by which vegetation contributes to health include moderation of ambient temperature, absorption of air pollution, and providing opportunity for physical activity (Nieuwenhuijsen et al., 2014).

Asthma is the most prevalent child respiratory disease, affecting 235 million people worldwide (World Health Organization [WHO], 2017) and 24.6 million Americans, including 6.2 million children (CDC, 2017). In the United States, costs of healthcare, lost school time for children, and lost work time for caregivers add up to $56 billion annually (Children’s Hospital Colorado, 2019). In 2013, 1.6 million visits to emergency departments in the United States were attributed to asthma exacerbation, and over 3,600 Americans died from asthma in 2015 (CDC, 2017). Because asthma is exacerbated by environmental exposures, it remains important for public health nurses to explore modifiable factors to improve outcomes for those affected, including characteristics of the physical environment such as greenness.

While the health benefits of greenness may be numerous, findings related to child asthma are mixed. A systematic review and meta‐analysis by Lambert et al. (2017) examined evidence regarding the relationship between greenness and allergic respiratory disease and asthma in children. Studies included examining asthma as the outcome demonstrated inconsistent results. Sbihi, Tamburic, Koehoorn, and Brauer (2015) found an inverse relationship between greenness and asthma for preschool children but found no relationship among school‐aged children. Lovasi, Quinn, Neckerman, Perzanowski, and Rundle (2008) found decreased asthma among children living in areas with more trees, but Lovasi et al. (2013) found increased greenness around the home significantly related to increased asthma at age seven. Andrusaityte et al. (2016) found greenness detrimental to child asthma, while others found no statistically significant relationship (Dadvand et al., 2014; Pilat et al., 2012). As considerations for future work, Lambert et al. (2017) discussed heterogeneity of findings and inconsistency in operational definitions of greenness, seasonal variation and proximity of greenness to be measured, and confounders such as proximity to high‐traffic roads and urban versus rural areas. Meta‐analysis added further to the body of evidence for discerning the relationship between greenness and child asthma, and additional studies have emerged since the review was published in 2017. The purpose of this literature review is to evaluate literature regarding greenness and asthma in children published since 2017, and to consider implications of this relationship for public health nursing.

## 2 METHODS

### 2.1 Design

**Figure 1:**
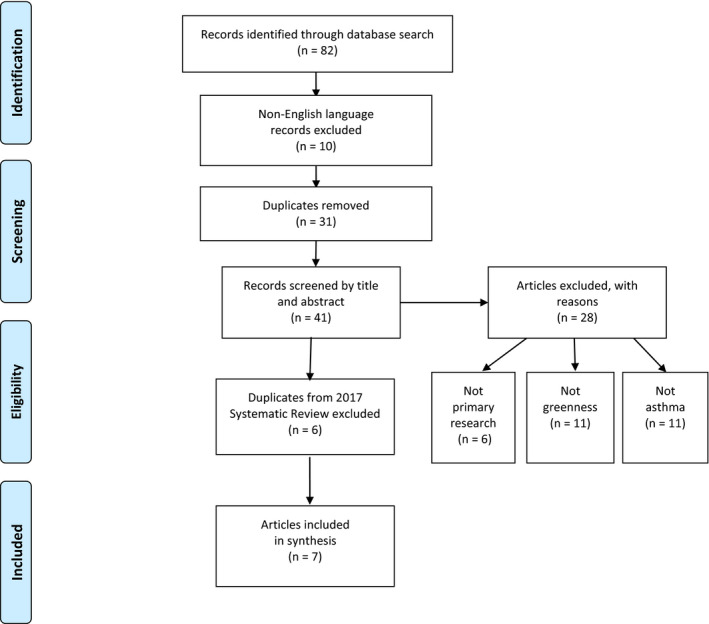
PRISMA Flow Diagram depicting literature search results (adapted from Moher et al., 2009) [Color figure can be viewed at wileyonlinelibrary.com]

This review was conducted following the PRISMA Statement (Moher, Liberati, Tetzlaff, & Altman, 2009). Reporting of the literature review meets criteria of the PRISMA 2009 Checklist. Search results are presented in the PRISMA Flow Diagram (Figure 1).

Search strategy included six databases: CINAHL with Full Text, Environment Complete, GreenFILE, Medline with Full Text, PsycINFO, and Urban Studies Abstracts. Given the interdisciplinary science related to greenness and health, these databases were chosen for results relevant to both health and environment. The search string was developed through repeated attempts to isolate terms specific to the constructs of greenness and asthma, using MeSH headings, searching known literature for keywords, and using truncation symbol (*) to search for all versions of the root word. The final search string included: [("greenspace*" OR "greenness" OR "green space*" OR "tree canopy" OR "vegetation" OR “street tree*” OR “urban forest*") AND ("asthma*" OR "respiratory" OR “lung function”) AND ("child*" OR "teen*" OR "adolescen*" OR "youth") in the abstract.

Inclusion criteria were international publications of primary research results relevant to the relationship between asthma and measured greenness in children under age 18, written in English language. International studies were included, as research related to greenness is emerging globally. Studies included in the Lambert et al. (2017) review were excluded.

### 2.2 Analytic strategy

Data were evaluated using created tables. Based on suggestions to standardize measurement of greenness (Lambert et al., 2017), conceptual, and operational definitions of exposures and outcomes are provided in Table 1. Study objective, characteristics, methods, and findings of each study were considered separately (Table 2).

**Table 1:** Definitions and measurement of exposures and health outcomes

Authors (Year)	Exposure and terms	Greenness measurement	Setting	Outcome variable	Outcome measurement
Chen et al. (2017)	Greenspace around the home; “greenspace” in title, abstract, and text	NDVI at 250 m radius, averaged seasonally	Urban Chicago, USA	Control of current asthma	Questionnaires to assess control of symptoms & functional limitations; clinical tests for airway inflammation and stress response (blood test).
Cillufo et al. (2018)	Greenness around the home; “greenness” in title, keywords, and abstract; “vegetation” in text	NDVI at 200 m (single pixel) around home address on a single date during month outcomes were assessed.	Urban Palermo, Italy	Respiratory and allergic conditions, general symptoms.	Parent and child report; classified as ocular, allergy, pulmonary, or general symptoms.
Donovan et al. (2018)	Natural environment, NDVI and other land cover types; “vegetation” in title, abstract and text; “greenness” in title & text	Max annual NDVI per meshblock; Land cover data for number of land cover types, native land cover types, & nonnative land cover types.	New Zealand	Asthma	ICD−10 code for asthma or ≥ 7 prescriptions for inhaled corticosteroids or inhaled beta‐adrenoceptor agonist between age 7–18.
Eldeirawi et al. (2019)	Residential surrounding greenness; “greenness” in title, abstract, and text	NDVI at 100 m, 250 m, 500 m on single date for theoretical maximum greenness	Urban and Suburban Chicago, United States	Lifetime asthma, lifetime wheeze, current wheeze, current dry cough at night.	Parent questionnaire used in Study of Asthma in Children of Mexican Descent (no tool specified).
Feng and Astell‐Burt (2017)	Exposure to publicly accessible green space; “green space”	% land use classified as “parkland”, stratified into 0%–20%, 20%–40%, and > 40%	Nationally representative sample in Australia	Affirmative asthma	Self‐report of physician diagnosis, asthma medication in the past 12 months, and wheezing for at least one week in past 12 months
Lambert et al. (2017)	Residential greenness; “greenness” in title, abstract, and text	NDVI at 100m	*N*/A	Asthma	Dichotomized as asthma yes/no
Tischer et al. (2017)	Residential surrounding greenness, proximity to green space, and surrounding greyness; “greenness” and “green space” used differently	Surrounding greenness measured as NDVI within 300m radius of residence at birth and age 4 years; proximity to green space using Urban Atlas map for green space (300 m radius).		Asthma, bronchitis, or allergic rhinitis	Age 1 year: asthma as wheezing ever, physician diagnosis of bronchitis Age 4 years: asthma as wheezing in past 12 months, physician diagnosis of bronchitis, allergic rhinitis in past 12 months.

Abbreviation: NDVI, Normalized Difference Vegetation Index.

**Table 2:** Characteristics of included studies

Authors (Year)	Study objective	Design & sample	Study methods	Relevant findings
Chen et al. (2017)	Investigate whether living in areas high in greenness may help buffer effects of difficult family relationships for children with asthma	Cross‐sectional; 150 children age 9–17 years in Chicago who were diagnosed with asthma in 2013–2014	Hierarchical multiple regression to predict asthma measures from demographic covariates, main effects of greenness and family relationships (measured as maternal hostility), and interaction between greenness and family relationships	No main effects of NDVI (250m) on asthma control [B = 0.05, 95% CI (−9, 17), *p* = .53] or functional limitations [B = 0.02, 95% CI (−4, 5), *p* = .84]. Interaction effect of greenness x parent hostility significant for asthma functional limitations [B= −0.14, 95% CI (−2, −0.05), *p* = .04] but not for asthma control [B = 0.03, 95% CI (−2, 3), *p* = .71]
Cillufo et al. (2018)	Evaluate association between urban environmental exposures to greenness, grayness, and NO2 air pollution with respiratory and allergic symptoms	Cross‐sectional; 219 schoolchildren age 8–10 in Palermo, Italy in April 2013.	Respiratory symptoms per parent and child questionnaire; NDVI; Land cover use for greyness; NO2 concentration from land use regression model	Participants lived near each other, so little variation in NDVI. No statistically significant relationships between NDVI ≤ 0.15 (1st quartile) and pulmonary symptoms (breathlessness, wheeze) [aOR = 0.98 95% CI (0.79, 1.21)].
Donovan et al. (2018)	Assess association between natural environment and asthma in children	Cohort; 49,956 children in New Zealand (1998–2016)	Used national database to assess outcomes, analyzed via three‐staged modeling approach (NDVI only, NDVI + # land cover types, NDVI + # and type of land cover)	1 *SD* increase lowers asthma risk for mean lifetime NDVI [OR = 0.92, 95% CI (0.89, 0.95), *p* < .01) and mean lifetime # land cover types [OR = 0.93, 95% CI (0.89, 0.99), *p* < .05); nonnative species increased asthma risk.
Eldeirawi et al. (2019)	Examine association between respiratory symptoms and residential surrounding greenness in urban children	Cross‐sectional; 1,915 children of Mexican‐American heritage in urban Chicago, IL in 2004	Multi‐level, mixed‐effect multiple regression used to determine association between greenness and parent‐reported respiratory symptoms.	NDVI at all buffers associated with lower odds lifetime asthma for those exposed to smoke [100 m: aOR 0.43 (95% CI: 0.22–0.87); 250 m: aOR 0.39 (95% CI: 0.18–0.84); aOR 0.48 (95% CI: 0.26–0.9)]; median NDVI slightly higher for nonasthmatics at all buffers but not significant.
Feng and Astell‐Burt (2017)	To investigate whether green space lowers child asthma risk by buffering effects of heavy traffic and a lack of neighborhood safety	Cross‐sectional; 4,447 children age 6–7 years old in Australia in 2006	Cross‐tabulations used to pattern asthma cases with respect to green space quantity, and perception of heavy traffic and area safety	Living near high traffic and low greenness had higher risk of asthma (OR 1.87, 95% CI: 1.37–2.55); living in high traffic and high greenness had lower risk (OR 0.32, 95% CI: 0.12–0.84).
Lambert et al. (2017)	Systematic review and meta‐analysis of residential greenness and allergic respiratory disease in children	Meta‐analysis of three studies	Threshold of 3 studies with same outcome and exposure in same buffer; random effects used; heterogeneity set at < 80%	No significant overall association (pooled OR 1.01, CI: 0.93–1.09; I^2^ 68%, *p* = .02)
Tischer et al. (2017)	Assess effect of three indices of urban built environment on allergic and respiratory conditions	Cohort; 2,472 children age 4 years from two distinct regions of Spain: Euro‐Siberian and Mediterranean	Longitudinal assessments at 1 year of age to assess asthma and bronchitis and 4 years of age to assess asthma, bronchitis, and allergic rhinitis	No significant relationships between greenness, proximity to green space, or greyness with any health outcome; Prevalence of asthma higher in Euro‐Siberian (rural) region (5%) than Mediterranean (urban; 2%); adjusted OR for asthma higher in 3rd tertile NDVI versus. 1st for cohort (aOR 1.82; CI: 0.71–4.67), Euro‐Siberian region (aOR 2.26; CI: 0.91–5.67), and Mediterranean region (aOR 2.05; CI: 0.69–6.06); proximity to green space protective for asthma but not significantly

Abbreviations: NDVI, Normalized Difference Vegetation Index; NO2, Nitrogen dioxide.

## 3 RESULTS

Initial search yielded 82 results. Removal of 31 duplicates and ten articles not in English language left 41 results. Title and abstract review excluded 28 articles that did not fit the research purpose: six were not primary research, 11 were not related to asthma, and 11 were unrelated to measured greenness. Although all publication dates were included in the search, no results appeared before 2017 that were not included in the review by Lambert et al. (2017). Of 13 remaining results, six were included in the previous review and excluded here to prevent over‐representation of findings. In total, seven articles were included in this analysis, consisting of six primary research studies and one systematic review with meta‐analysis.

The grading system used for this review was the Johns Hopkins Research Evidence Appraisal tool, part of the Johns Hopkins Nursing Evidence‐Based Practice model. The goal of the model is to ensure research findings and best practices are quickly and appropriately incorporated into patient care (Dang & Dearholt, 2017). Because all included articles were observational, all were graded at level III.

### 3.1 Terminology

Several terms are used to describe the concept of greenness, including “greenness,” “green space,” “greenspace,” and “vegetation.” Frequency table of terms used in included studies is provided in Table 3.

**Table 3:** Frequency table of terms

	Greenness	Greenspace	Green space	Vegetation
Title	4	2	1	1
Abstract	4	2	1	1
Keywords	1	1	0	0
Text	4	2	1	2

### 3.2 Measurement of greenness

Methods for measuring greenness differed across studies. A common measurement is Normalized Density Vegetation Index (NDVI), calculated from visible and near‐infrared light reflected by vegetation, compiled through satellite imagery. NDVI values range from −1 to + 1, with negative values representing features such as water, values near zero representing no vegetation, and values approaching + 1 indicating the most greenness (National Aeronautics & Space Administration [NASA], 2018; Sentinel, n.d.). NDVI provides free and publicly available data for measuring greenness, which can aid replication and consistency across studies (Rhew, Vander Stoep, Kearney, Smith, & Dunbar, 2011). This convenience may account for more frequent use of NDVI as a measurement of greenness in the included studies.

Six of the seven studies in this review used NDVI for at least one measure of greenness (Chen et al., 2017; Cillufo et al., 2018; Donovan, Gatziolis, Longley, & Douwes, 2018; Eldeirawi et al., 2019; Lambert et al., 2017; Tischer et al., 2017). Of those six, two combined NDVI with land‐use classification as another measure of greenness: total and native land‐use types (Donovan et al., 2018) and urban green areas (Tischer et al., 2017). Feng and Astell‐Burt (2017) did not use NDVI but used land classified as “parkland”.

Average NDVI is calculated within a circular radius (buffer) from a specified point, usually home address. Among the six studies using NDVI, four examined only one buffer distance: 100m (Lambert et al., 2017), 200m (Cillufo et al., 2018), 250m (Chen et al., 2017), and 300m (Tischer et al., 2017). One study used nested buffers of 100m, 250m, and 500m (Eldeirawi et al., 2019), a practice suggested for approximating greenness near the home, neighborhood, and larger community. One article did not specify buffer, but used meshblocks, statistical subdivisions not comparable to buffer radii as they are nonuniform in size (Donovan et al., 2018; Stats NZ, n.d.).

### 3.3 Greenness and asthma

Out of the seven reviewed papers, six reported no statistically significant direct relationships between greenness and child asthma while one (Donovan et al., 2018) found 1 standard deviation increase in NDVI was associated with a 6% lower risk of asthma (95% CI: 1.9%‐9.9%).

Three papers reported greenness was protective for child asthma via mediation of other negatively related health factors, such as difficult family relationships (Chen et al., 2017), high traffic volume (Feng & Astell‐Burt, 2017), and tobacco smoke exposure (Eldeirawi et al., 2019). While Chen et al. (2017) reported no main effects of greenness on asthma control or functional limitations of children with asthma, interaction effect of greenness x parent hostility showed an inverse relationship protective for functional limitations of children with asthma. Feng and Astell‐Burt (2017) reported no association between greenness and asthma incidence for children not exposed to heavy traffic, but children living in areas of both high traffic and low greenness (characterized as 0%–20% land coverage) had higher risk of asthma (OR 1.87, 95% CI: 1.37–2.55) and those living in high traffic and high greenness (>40% land coverage) had lower risk (OR 0.32, 95% CI: 0.12–0.84). Eldeirawi et al. (2019) reported for children exposed to tobacco smoke, NDVI at all buffers was associated with lower odds for lifetime asthma [100 m: aOR 0.43 (95% CI: 0.22–0.87); 250 m: aOR 0.39 (95% CI: 0.18–0.84); 500 m: aOR 0.48 (95% CI: 0.26–0.9)] and median NDVI was slightly higher for nonasthmatics at all buffers, but did not reach statistical significance.

Cillufo et al. (2018) and Tischer et al. (2017) also found no direct relationship between greenness and child asthma, but unlike studies by Chen et al. (2017), Feng and Astell‐Burt (2017), and Eldeirawi et al. (2019), greenness was not presented as a mediator of other factors contributing to child asthma. Lack of a direct association between greenness and child asthma was supported in a 2017 systematic review and meta‐analysis by Lambert et al., who found no direct relationship between greenness and child asthma (pooled OR 1.01, 95% CI: 0.93–1.09). However, in that analysis, only three studies reported NDVI in the same buffer (100 m) to qualify for meta‐analysis (I^2^ = 68%, *p* = .02). This supports the suggestion that standardization of NDVI measurement would aid in future meta‐analyses (Lambert et al., 2017).

## 4 DISCUSSION

In this emerging area of scientific interest, greenness is operationalized differently among studies. Two articles in this analysis differentiated between types of greenness exposures within their study. Tischer et al. (2017) defined nondifferentiated vegetation around the home as “residential surrounding greenness,” quantified using NDVI, while proximity to “green space” was defined as home address within 300 m of a forest or park, measured using the Urban Atlas map of Europe. Donovan et al. (2018) measured “greenness” as NDVI and “vegetation diversity” as land cover types. Explicitly defining similar‐sounding variables within the same study is important for building knowledge on this emerging construct.

Lambert et al. (2017) suggested in their review of greenness and child respiratory health that future research should include standardized greenness measurement to aid in synthesis of findings. Results of this review demonstrate this recommendation has not been met. Although six of seven studies included here used NDVI, no two articles shared the same single buffer size and only one study employed concentric buffers to approximate greenness in the immediate area around the home, in the neighborhood, and at the community level. Such inconsistency precluded meta‐analysis in this review, prohibiting a higher level of statistical evidence for drawing more robust conclusions about the effect of greenness on child asthma.

Studies in this review acknowledged difficulty in isolating effects of greenness from multiple confounders, particularly related to socioeconomic status. Associations often lost statistical significance after adjusting for individual‐level confounders such as maternal education, household income, and second‐hand smoke exposure. Eldeirawi et al. (2019) suggest in their discussion that their finding related to second‐hand smoke exposure may be a proxy for low socioeconomic status. Two studies (Chen et al., 2017; Cillufo et al., 2018) only included individual‐level confounders in analysis, neglecting the effect of community. Because asthma is affected by both individual and community‐level factors, it is important to consider influences of both categories for isolating the effect of greenness.

There is growing interest in understanding how greenness can be leveraged to support public health, but there are limited published findings. Terms used to refer to the concept of greenness vary. In these results, terms included “greenness,” “green space,” “greenspace,” and “vegetation” (Table 3). Other terms in the study of greenness may refer to trees, such as “street trees,” “urban forest,” “tree cover,” and “tree canopy.” Such variation in terminology may complicate database searches, inhibiting communication of findings. When conducting literature searches regarding greenness and health, public health nurses should search across disciplines and include multiple search terms.

Two articles relay contradictory findings for asthma in children living in rural versus urban areas. Donovan et al. (2018) reported children who had always lived in rural New Zealand had lower odds of asthma (OR 0.85, 95% CI: 0.74–0.99). Comparing 2,472 children in rural versus urban Spain, Tischer et al. (2017) found higher prevalence of asthma in rural Euro–Siberian region (5%) versus more urban Mediterranean region (2%). In their 2012 literature review, Malik, Kumar, and Frieri (2012) found minimal difference in prevalence of asthma in urban versus rural environments, suggesting risk factors and socioeconomic trends in both settings create need for individual‐level and community‐level prevention efforts. The effect of environmental greenness is therefore an important point of consideration for public health nurses working in both urban and rural settings.

Among seven studies reviewed, six reported no direct association between greenness and child asthma, while one reported increased greenness protective of asthma. When findings of this review are considered with findings of the review by Lambert et al. (2017), who found two articles showing benefit of greenness for child asthma, two articles showing a detrimental relationship, and four articles showing no relationship, it seems a direct relationship either does not exist or has not been accurately measured. No studies included here found a negative direct association between greenness and child asthma. There was, however, evidence to support greenness mediates negative effects of other factors that may directly affect child asthma.

### 4.1 Implications for public health nursing

This review found either positive or no direct association between greenness and child asthma. There was no indication greenness is detrimental to child asthma, and it may mediate factors contributing to child asthma. Together with evidence supporting the relationship between greenness and multiple health outcomes, these findings indicate greenness can be considered a community asset. As public health nurses assess risk in geographic areas and capture population assets (ANA, 2007), greenness should be included in such assessments as communities lacking greenness may be at risk for poor health outcomes.

In order to promote greenness as a community asset, public health nurses can support programs to protect and increase greenness. Arbor Day Foundation (2019), for example, offers programs such as “Tree City USA” and “Tree Cities of the World” to encourage growth and maintenance of urban tree canopy. Using tools provided by the United States Department of Agriculture's Forest Service (2019), public health nurses can assess existing tree canopy to determine need for greenness in communities served and partner with communities to organize tree planting programs. Public health nurses can identify areas that would support gardens or expanded tree coverage, such as schools, parks, playgrounds, street medians, and parking lots. Through greening initiatives, public health nurses can provide assets to build and protect community health. Partnering with local politicians, public health nurses can advocate for policies that expand and protect greenness.

## 5 CONCLUSION

Greenness has no direct effect on child asthma but may be protective via modification of individual and community‐level risk factors. Results of this updated review are consistent with a prior systematic review. Further, this review found a protective effect of greenness through moderation of factors contributing to child asthma, such as difficult family relationships, high traffic volume, and tobacco smoke exposure. Public health nurses should share these findings with individual and community partners, consider greenness a community asset for promoting health, and advocate for policies and programs to increase and protect existing greenness.
